# Preconcentration and Determination of Perfluoroalkyl Substances (PFASs) in Water Samples by Bamboo Charcoal-Based Solid-Phase Extraction Prior to Liquid Chromatography–Tandem Mass Spectrometry

**DOI:** 10.3390/molecules23040902

**Published:** 2018-04-14

**Authors:** Ze-Hui Deng, Chuan-Ge Cheng, Xiao-Li Wang, Shui-He Shi, Ming-Lin Wang, Ru-Song Zhao

**Affiliations:** 1 College of Food Science and Engineering, Shandong Agricultural University, Taian 271018, China; dengzh940209@126.com; 2 Key Laboratory for Applied Technology of Sophisticated Analytical Instruments of Shandong Province, Analysis and Test Center, Qilu University of Technology (Shandong Academy of Sciences), Jinan 250014, China; chengchg@sdas.org (C.-G.C.); zhaors1976@126.com (R.-S.Z.); 3 Environmental Monitoring Station of Dongming Environmental Protection Bureau, Dongming 274500, China; 18354080666@163.com

**Keywords:** bamboo charcoal, solid-phase extraction, perfluoroalkyl acids, liquid chromatography–tandem mass spectrometry

## Abstract

In this work, bamboo charcoal was used as solid-phase extraction adsorbent for the enrichment of six perfluoroalkyl acids (PFAAs) in environmental water samples before liquid chromatography–tandem mass spectrometry analysis. The specific porous structure, high specific surface area, high porosity, and stability of bamboo charcoal were characterized. Several experimental parameters which considerably affect extraction efficiency were investigated and optimized in detail. The experimental data exhibited low limits of detection (LODs) (0.01–1.15 ng/L), wide linear range (2–3 orders of magnitude and R ≥ 0.993) within the concentration range of 0.1–1000 ng/L, and good repeatability (2.7–5.0%, *n* = 5 intraday and 4.8–8.3%, *n* = 5 interday) and reproducibility (5.3–8.0%, *n* = 3). Bamboo charcoal was successfully used for the enrichment and determination of PFAAs in real environmental water samples. The bamboo charcoal-based solid-phase extraction coupled with liquid chromatography–tandem mass spectrometry analysis possessed great potential in the determination of trace PFAA levels in environmental water samples.

## 1. Introduction

Perfluoroalkyl Substances (PFASs) consists of a C–F bond, which is one of the strongest chemical bonds; these compounds are both hydrophobic and lipophobic [1]. Fluorine is the most electronegative element, and the unique physical and chemical properties of perfluorinated organic compounds can be achieved by the introduction of fluorine atoms. Perfluorinated organic compounds, which exhibit good chemical stability, outstanding surface activity, and excellent thermal stability, are extensively applied in cutting-edge technologies, major industrial projects, pharmaceuticals, pesticides, and other industries. PFASs are distributed in various samples, such as water [2,3,4,5], soil and sediments [6,7,8,9], biological samples [10], and food samples [11] because of their high stability. The hazards of these organic compounds have been reported recently. The Organization for Economic Cooperation and Development and the US Environmental Protection Agency classified PFASs as “potentially carcinogenic substances”. These compounds have attracted considerable attention worldwide. Improved methods are needed to be sought for monitoring of slow PFAS levels in a variety of samples.

Many organic contaminants have been detected at trace levels in recent years because of the coupling of gas or liquid chromatography (LC) with mass spectrometry (MS) techniques [12,13]. LC coupled with tandem MS (LC-MS/MS) is an effective analytical method for the sensitive and selective detection of PFASs [14,15,16]. Direct analysis of PFASs is almost impossible because of their ultra-low concentration in various samples and the complexity of sample matrices [14]. A simple, convenient, time-saving, and solvent-free sample pretreatment technique prior to LC-MS/MS analysis is required.

Sample processing techniques, including pressurized-liquid extraction (PLE) [17], solvent extraction [4], dispersive solid-phase extraction [18,19,20], solid-phase extraction (SPE) [21,22,23], magnetic solid-phase extraction (MSPE) [24,25,26] and other techniques, are utilized to enrich trace PFAS levels in environmental and biological samples prior to chromatographic analysis. Organic solvents used in PLE are toxic to the environment, and this technique is time consuming. In the 1970s, SPE technology has replaced traditional liquid–liquid extraction as an effective pretreatment method. SPE technology has been widely used in food, biological, pharmaceutical, and environmental analyses because of its reliability, high efficiency, simple operation, and low solvent consumption [27,28]. Traditionally, C18, oasis WAX sorbent, and HLB polymer were used as SPE sorbents to enrich PFCs in biological and environmental samples [29,30,31,32]. Bamboo charcoal, a new biomaterial with special microporous characteristics, has attracted great attention in many fields in recent years. Bamboo charcoal has been widely used for the enrichment of pollutants in environmental samples because of its relatively low price, specific porous structure, high porosity, and stability [33,34].

In this study, bamboo charcoal was used as a SPE sorbent to enrich six perfluoroalkyl acids (PFAAs) in water samples. The effects of bamboo charcoal on the experimental parameters, such as eluent, eluent flow rates, pH, sample volume, and eluent volume, were evaluated, and the parameters on extraction efficiencies were optimized. A simple, low-cost, and highly selective and sensitive SPE-HPLC-MS/MS method was established and applied for the sensitive determination of PFASs in environmental water samples.

## 2. Results and Discussion

### 2.1. Characterization of Bamboo Charcoal

**Figure 1:**
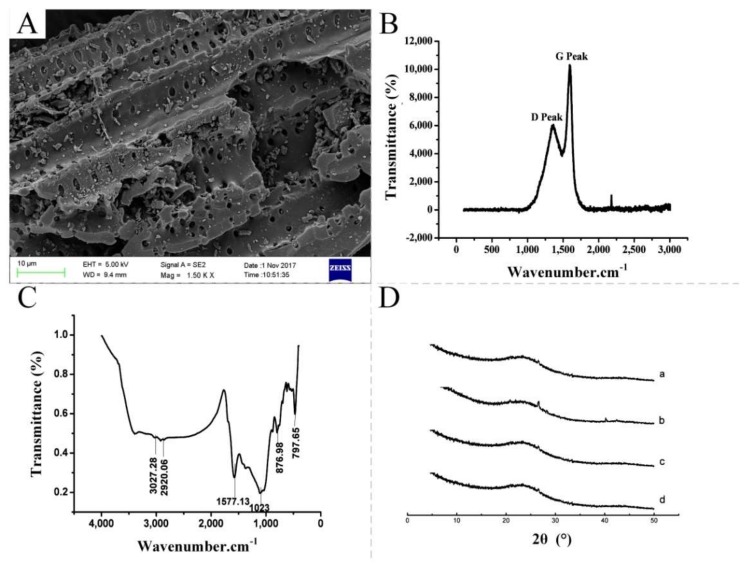
(**A**) SEM image of the bamboo charcoal at 1,500× magnification; (**B**) Raman spectra of the bamboo charcoal; (**C**) FTIR spectra of the bamboo charcoal; and (**D**) XRD patterns of the bamboo charcoal in: air (a); HCl aqueous solution, pH 2 (b); NaOH aqueous solution, pH 12 (c); and methanol for 24 h (d).

A SEM micrograph of the bamboo charcoal is shown in Figure 1A, and the porous structure of the material can be seen clearly. The BET-specific surface area of the bamboo charcoal was 31.932 m^2^/g. Bamboo charcoal can be used as an effective sorbent for environmental pollutants because of its plentiful cavity construction and high specific surface area.

A Raman spectrum of the bamboo charcoal is shown in Figure 1B. The peak positions of D and G were determined by the mechanical constants of C–C bonds in the carbon network plane of the graphite microcrystal or graphite-like microcrystal. Various oxygen-containing functional groups were present at the edge of the graphite-like microcrystal that formed in the low-temperature carbonization stage of biomass carbon. Ether bonds may also be present between the monolayer carbon planes of the graphite-like microcrystals. The existence of these functional groups or bonds may affect the delocalized π electron behavior in the carbon network plane. Thus, the mechanical constants of the C–C bond were increased or decreased, and Raman shifts were detected. The D peak was caused by the sp2-hybridized-carbon atoms at the edge of the graphite microcrystal, and the G peak was caused by the translational vector of the symmetrical structure in the carbon network plane of the graphite microcrystal. Thus, the oxygen-containing functional groups between the carbon network and those at the edge of the carbon network exhibited different effects on their Raman spectra.

As shown in the FTIR spectra (Figure 1C), the bamboo charcoal exhibited an absorbance peak at approximately 1577.13 cm^−1^ due to the stretching vibration of the carbonyl group (C=O). Two absorbance peaks at approximately 797.65 and 1023 cm^−1^ were assigned, respectively, to the out-of-plane bending vibration of the C–H group and stretching vibrations of the C–O and C–O–O–C groups. These results verified the existence of a carbonyl group (C=O) on the bamboo charcoal. Considering that the electronegativity of an oxygen atom (3.5) is higher than that of a carbon atom (2.5), the electron cloud distribution of the C=O bond is biased toward the oxygen atom, which determines polarity and chemical reactivity with numerous polar substances of the C=O group. PFCs are a group of environmental organic pollutants with strong polarity, and the C=O group on the bamboo charcoal can react with PFCs, supporting bamboo charcoal as a novel SPE sorbent for sensitive PFC extractionin environmental water samples.

The chemical stability of the bamboo charcoal in extreme conditions, such as acidic, alkaline, and organic solvents, was investigated in this study. The bamboo charcoal (500 mg) was immersed separately in NaOH solution (pH = 13), HCl solution (pH = 2), and methanol at room temperature for 24 h. As shown in Figure 1D, no evident changes in the XRD patterns were observed under different experimental conditions. These results indicated that bamboo charcoal is stable in aqueous solution with a broad pH range of 2–13 and organic solvents and is suitable for environmental pollutant analysis.

### 2.2. Optimization of the Experimental Parameters

To acquire optimized extraction conditions, effective parameters, such as the eluent, eluent volume and flow rate, sample pH, and sample volume and flow rate, were investigated and optimized in detail. In this work, 100 mL of ultrapure water spiked with 10 μg/LPFAAs (PFHxS, PFHpA, PFOA, PFOS, PFNA, and PFDA) was used to investigate the SPE performance of bamboo charcoal.

**Figure 2:**
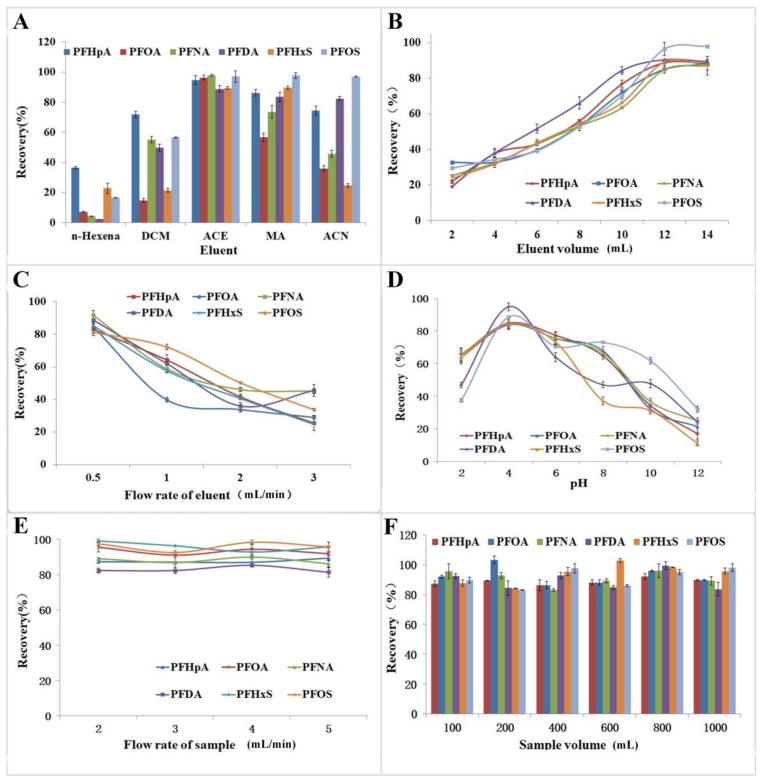
Effects of the: eluent (**A**); eluent volume (**B**); flow rate of eluent (**C**); pH (**D**); flow rate of sample (**E**); and sample volume (**F**) on the recoveries of the six PFAAs. The PFAA concentration in the water samples was 100 ng/L.

The eluent is one of the most important factors in sample preconcentration procedure. In this experiment, the solvents acetone, methanol, acetonitrile, dichloromethane, and *n*-hexane were tested. The desorption efficiency of these five solvents are shown in Figure 2A. Acetone exhibited the best elution performance for the PFAAs among the five studied solvents. Therefore, acetone was chosen as the desorption solvent in subsequent work.

The influence of eluent (acetone) volume (2–14 mL) on the desorption efficiency of PFAAs was examined (Figure 2B). The desorption efficiencies increased as the eluent volume increased from 2 mL to 12 mL. The desorption efficiency did not significantly increase at >12 mL elution volumes. Thus, 12 mL of acetone as the eluent volume was adopted in the following experiments.

Eluent flow rate is also an important factor that affects desorption efficiency because it influences the contact time between the molecules of target pollutants and the eluent [35]. The eluent flow rate was investigated and optimized at 0.5, 1, 2, and 3 mL/min to save desorption time and obtain satisfactory results (Figure 2C). The recoveries of the six PFAAs increased with decreasing flow rate. Thus, the flow rate of 0.5 mL/min was chosen for subsequent analytical experiments.

The influence of pH (2.0–12.0) on the extraction efficiency was investigated. The recoveries reached the optimal level at pH 4.0 (Figure 2D). These results illustrated that PFAAs can be effectively adsorbed onto the bamboo charcoal sorbents under acidic conditions. The possible reason is that the pH of the sample solution affects the forms of PFAAs existing in the aqueous samples. Under acidic conditions, PFAAs mainly exist as unionized acid, whereas, under neutral or alkaline conditions, PFAAs are mainly ionized and soluble in water samples, leading to decreased adsorptive efficiency from water samples to adsorbents [27]. Thus, the sample pH was adjusted to 4.0 in subsequent experiments.

The flow rate of 2–5 mL/min was investigated to save analytical time and obtain satisfactory experimental results (Figure 2E). At 2–5 mL/min flow rates, the recoveries obtained were between 81.39% and 99.12%. The sample flow rate of 5 mL/min was selected for the subsequent experiments of the PFAAs. Sample volume was also optimized in the experiment. The recovery remained stable when the sample volume increased from 100 mL to 1000 mL (Figure 2F), and the reactions between the targeted pollutants and bamboo charcoal were not affected by the sample volume. The sample volume of 100 mL was selected for the subsequent experiments.

### 2.3. Method Evaluation

The analytical data for the six kinds of PFAAs using bamboo charcoal as SPE adsorbent under optimal parameters are summarized in Table 1. The developed method exhibited good linearity (R ≥ 0.993) within the concentration range of 0.1–1000 ng/L. The limits of detection (LODs) based on signal-to-noise (S/N) ratios of 3 ranged from 0.01 ng/L to 1.15 ng/L. The limits of quantification (LOQs), which is calculated by S/N ratios of 10, ranged from 0.03 ng/L to 3.85 ng/L. The relative standard deviations (RSDs) of the intraday (*n* = 5) and interday (*n* = 5) experiments when using bamboo charcoal as the SPE adsorbent coupled to LC-MS/MS were in the range of 2.7–5.0% and 4.8–8.3%, respectively, for the six kinds of PFAAs. This finding illustrated the good repeatability and reproducibility of this method by using a single SPE column. Three SPE columns were prepared under the same conditions, and the column-to-column reproducibility (*n* = 3) was 5.3–8.0% for the six PFAAs (100 ng/L). As shown in Table 2, this method produced a wider linear range, lower LODs and LOQs, and higher accuracy efficiency compared with other methods mentioned in previous studies [20,24,31,32,36]. Moreover, one bamboo charcoal column can be reused more than 10 times without a detectable extraction efficiency loss. The experimental data exhibited that bamboo charcoal is suitable as a novel extraction adsorbent for the analysis of strong polar PFAAs in environmental samples.

### 2.4. Analysis of Fortified Samples for Recoveries Calculation

**Figure 3:**
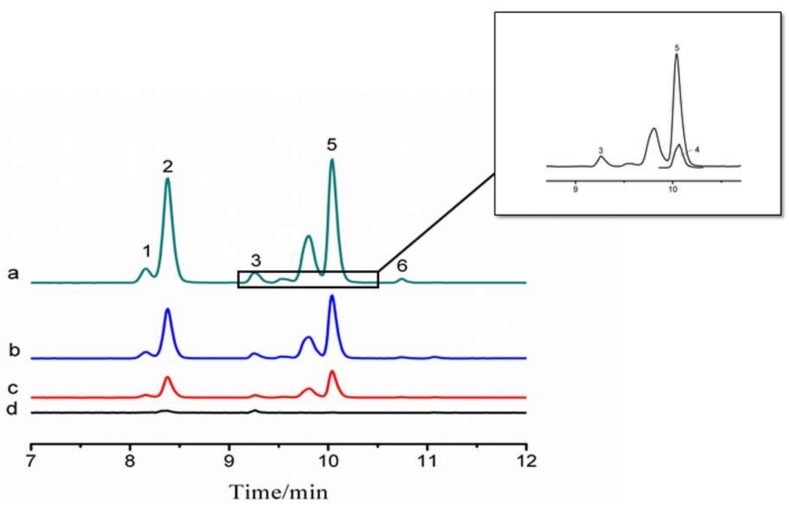
Typical chromatograms of the six PFAAs in real water samples. Pond water spiked at: (a) 100; (b) 50; and 20 ng/L (c); and pond water (d). (1) PFHpA; (2) PFHxS; (3) PFOA; (4) PFNA; (5) PFOS; and (6) PFDA.

The proposed SPE method with bamboo charcoal as adsorbent was then applied to analyze PFAAs in four real water samples, namely, barreled drinking water, tap water, pond water, and water collected from Dagu Port Scenic Resort. As shown in Table 3, PFHxS was detected at 0.56 ng/L in the tap water samples, and both PFHxS and PFOA were detected at 4.61 and 3.93 ng/L in pond water samples, respectively. No PFAA pollutants were detected in the barreled drinking water samples and water samples collected from Dagu Port Scenic Resort. Recovery testing was performed by spiking three different levels of PFAAs (20, 50, and 100 ng/L) in the four samples. The recoveries were within 86.9–117.2% at 0.4–8.3% RSDs. Typical chromatograms of PFAAs in an environmental water sample are illustrated in Figure 3. We can conclude from all of the experimental data that the analytical method established in this work is suitable for the analysis of PFAAs at trace levels in real water samples.

## 3. Materials and Methods

### 3.1. Chemicals and Reagents

Bamboo charcoal was purchased from Zhejiang Forasen Bamboo Tec Co., Ltd. (Zhejiang, China). The bamboo charcoal was first triturated in a glass mortar, sieved through an 80-mesh sieve, and dried at 80 °C for 2 h [27].

Perfluorohexanesulfonate (PFHxS), perfluoroheptanoic acid (PFHpA), perfluorooctanoic acid (PFOA), perfluorooctanesulfonic acid (PFOS), perfluorononanoic acid (PFNA), and perfluorodecanoic acid (PFDA) were purchased from AnpuShiyan Tech Co., Ltd. (Shanghai, China). Methanol, acetone, and acetonitrile were obtained from Tedia Company (Fairfield, OH, USA). Dichloromethane and *n*-hexane were purchased from Concord Technology (Tianjin, China). All other reagents and chemicals used in this experiment were of at least analytical grade. PFAS stock solution containing PFOA, PFHpA, PFNA, PFDA, PFHxS, and PFOS at 1 µg/mL was prepared by dissolving 0.1 mg of each of the six types of PFCs in a 100 mL volumetric flask. A series of standard solutions was obtained by gradually diluting the stock solution with methanol. All solutions were stored at 4 °C in the dark prior to use.

### 3.2. Instrument

In this work, a Thermo Ultimate 3000 Liquid Chromatograph (Thermo Scientific, Waltham, MA, USA) coupled with an AB SCIEX QTRAP 5500 triple quadrupole mass spectrometer (SCIEX, Framingham, MA, USA) was used. An Agilent XDB-C18 column (2.1 mm × 150 mm, 3.5 µm, Santa Clara, CA, USA) was used for the chromatographic separation at 40 °C. The mobile phases were 5 mmol/L NH4Ac (A) and methanol (B). The gradient elution during the chromatographic run was as follows: 0–1.0 min, 10% B; 1.1–1.5 min, 10–40% B; 1.6–12.0 min, 40–95% B; 12.1–13.0 min, 95% B; and 13.1–17 min, 10% B. The flow rate of the mobile phase was set at 0.4 mL/min, and the injection volume was 10 µL. The mass spectrometer analysis was conducted in the negative ionization mode with multiple reaction monitoring mode. The source temperature was 550 °C, and nitrogen was used as the collision gas. The ion spray voltage was −4500 V, the curtain gas was 40 psi, and the ion source gases 1 and 2 were 55 and 60 psi, respectively. The optimized MS/MS parameters are listed in Table 4.

The scanning electron microscopy (SEM) images of the bamboo charcoal were obtained using SUPPA™ 55 (Zeiss, Oberkochen, Germany). X-ray diffraction (XRD) measurements with the angle ranging from 10° to 50° were obtained with Cu Ka radiation on a D/max-Rbdiffractometer (Rigaku, Japan). The Brunauer–Emmett–Teller (BET)-specific surface areas of the bamboo charcoal were measured using an ASAP 2020 porosimeter (Micromeritics, Norcross, GA, USA). FTIR spectra were obtained using a Nicolet 710 IR spectrometer (Thermo Scientific, Waltham, MA, USA). The Raman spectrum of the bamboo charcoal was obtained using Renishaw inVia microscopes and a Raman spectrometer (Renishaw, Sheffield, UK).

### 3.3. SPE

Bamboo charcoal-packed cartridges were prepared based on previous literature [28,37]. Bamboo charcoal powder (300 mg) that was treated as mentioned above was packed in an empty SPE cartridge. The polypropylene frit was reset to hold the bamboo charcoal powder in place. The inlet of the cartridge was connected to a PTFE suction tube, which was inserted into the sample solution. The outlet of the cartridge was connected to a vacuum pump. The SPE cartridge was washed with purified water and acetone several times before its first use to reduce possible contaminants.

The bamboo charcoal column was washed and activated with 5 mL of purified water and 5 mL of acetone. Subsequently, 100 mL of water sample spiked with six PFAAs was passed through the pretreated cartridge at 5 mL/min. The cartridge was then rinsed with 10 mL of purified water to remove possible adsorbed matrix materials from the column. The bamboo charcoal column was then dried at negative pressure for 5 min. Subsequently, the target compounds retained on the bamboo charcoal were eluted with 12 mL of acetone, and the eluent was dried at 40 °C under nitrogen. Finally, the residue was dissolved in 1.0 mL of methanol prior to HPLC-MS/MS analysis.

### 3.4. Water Sample Collection

Four kinds of water samples, namely, barreled drinking water, tap water, pond water, and water collected from Dagu Port Scenic Resort, were used to evaluate the feasibility of the developed method. The barreled drinking water samples were obtained from the local supermarket (Jinan, China). The tap water samples were collected from our laboratory (Jinan, China). The pond water samples were acquired from the pond located at the Analysis and Test Centre (Jinan, China). After filtration through a 0.45 μm membrane filter, these water samples were stored in brown glass bottles at 4 °C for subsequent SPE extractions.

## 4. Conclusions

In this study, bamboo charcoal was used as an SPE adsorbent for the first time to enrich and analyze six kinds of new persistent organic pollutant perfluorooctanoic acids at trace levels in water samples. This novel adsorbent achieved good chemical stability; high repeatability, good reproducibility, and extraction efficiency; wide linear range (2–3 orders of magnitude); and low LODs (0.01–1.15 ng/L) for the analysis of PFAAs. An affordable and easily available material, bamboo charcoal is suitable as an SPE adsorbent for the extraction and analysis of polar organic pollutants in environmental water samples.

## Figures and Tables

**Table 1:** Analytical data of the SPE method.

Compounds	Linear Range (ng/L)	R	LODs (ng/L)	LOQs (ng/L)	Repeatability (%, *n* = 5)		Column-to-Column Reproducibility (%, *n* = 3)
					Intraday	Interday	
PFHpA	1.0–200.0	0.999	0.11	0.37	3.3	6.8	6.4
PFOA	1.0–200.0	0.999	0.07	0.22	2.7	5.4	7.3
PFNA	4.0–1000	0.999	1.15	3.85	3.6	4.8	7.8
PFDA	10.0–1000	0.997	0.88	3.68	4.1	8.3	8.0
PFHxS	0.1–100	0.993	0.01	0.03	5.0	5.1	5.8
PFOS	0.1–100	0.998	0.01	0.03	2.9	7.0	5.3

**Table 2:** Method comparisons for the analysis of the six PFAAs.

Material	Analytical Methods	Linear Range (ng/L)	LODs (ng/L)	RSD (%)	Recoveries (%)	References
Fe_3_O_4_@mSiO_2_-F17	MSPE-HPLC-MS/MS	250–1,000,000	20–50	2.6–14.2	83.13–92.42	[24]
C18, PSA, GCB	QuEChERS-HPLC-MS/MS	100–10,000	50–200	2.1–11.9	70.3–108.1	[20]
HLB	SPE-HPLC-MS	500–200,000	150–900	7.5–11.8	73–88	[31]
CTAB-MCM-41	μ-SPE-LC-MS	1000–100,000	970–2700	5.4–13.5	77–120	[32]
Octadecylsiyl particles	SPE-Reversed Phase-HPLC-MS	-	25	0.5–10.8	79.2–96.1	[36]
Bamboo charcoal	SPE-LC-MS/MS	0.1–1250	0.01–1.44	0.4–8.3	86.9–117.2	This work

PSA: *N*-propylethylendiamine; GCB: graphitized carbon blacks; HLB: The HLB adsorbent is a macroporous copolymer that is polymerized from lipophilic divinylbenzene and hydrophilic *N*-vinylpyrrolidone in a certa proportion; CTAB-MCM-41: a kind of new material (cetyltrimethylammonium bromide contained MCM-41); MSPE: magnetic solid phase extraction; QuEChERS: a quick, easy, cheap, effective, rugged and safe sample pretreatment method.

**Table 3:** Analytical results for the determination of the six PFAAs in real water samples.

Samples	Added (ng/L)	PFHpA	PFOA	PFNA	PFDA	PFHxS	PFOS
Barreled drinking water	0.0	ND ^a^	ND ^a^	ND ^a^	ND ^a^	ND ^a^	ND ^a^
	20.0	104.2 ^b^ ± 6.8 ^c^	102.8 ± 3.1	97.2 ± 1.3	102.4 ± 2.3	96.1 ± 4.1	92.0 ± 2.4
	50.0	94.3 ± 4.7	104.9 ± 4.2	96.0 ± 3.2	109.3 ± 1.8	100.5 ± 5.5	100.5 ± 3.1
	100.0	89.7 ± 7.0	99.7 ± 5.2	99.2 ± 1.6	100.3 ± 3.7	103.2 ± 3.9	96.8 ± 5.1
Tap water	0.0	ND ^a^	ND ^a^	ND ^a^	ND ^a^	0.56	ND ^a^
	20.0	95.4 ± 0.9	87.5 ± 6.3	90.6 ± 4.1	99.4 ± 8.3	99.1 ± 6.1	89.3 ± 2.9
	50.0	98.6 ± 1.4	94.6 ± 2.7	93.8 ± 2.3	95.3 ± 3.8	94.5 ± 5.1	93.2 ± 3.0
	100.0	111.4 ± 5.3	93.7 ± 2.3	98.5 ± 3.2	98.7 ± 6.2	91.2 ± 1.7	91.6 ± 1.4
Pond water	0.0	ND ^a^	3.93	ND ^a^	ND ^a^	4.61	ND ^a^
	20.0	92.8 ± 6.2	117.2 ± 3.8	103.7 ± 3.1	86.4 ± 0.9	83.4 ± 4.6	107.3 ± 6.9
	50.0	98.1 ± 7.4	105.6 ± 4.5	104.2 ± 2.4	89.3 ± 4.2	86.9 ± 3.1	105.3 ± 2.5
	100.0	107.3 ± 2.4	102.2 ± 4.1	101.3 ± 5.1	91.0 ± 1.7	84.1 ± 1.9	99.6 ± 3.4
Port water	0.0	ND ^a^	ND ^a^	ND ^a^	ND ^a^	ND ^a^	ND ^a^
	20.0	92.4 ± 1.4	98.4 ± 6.1	101.1 ± 0.4	85.4 ± 4.2	97.3 ± 0.4	93.2 ± 4.1
	50.0	87.3 ± 4.3	99.1 ± 4.3	100.3 ± 3.6	87.5 ± 1.3	91.4 ± 4.7	97.5 ± 7.3
	100.0	102.4 ± 5.1	92.9 ± 5.5	97.8 ± 6.7	93.6 ± 1.7	89.6 ± 2.9	91.4 ± 1.8

^a^ Not detected; ^b^ Mean value of three determinations; ^c^ Standard deviation (*n* = 3).

**Table 4:** HPLC–MS/MS parameters for MRM acquisition of PFAAs.

Compounds	Retention Time (min)	Precursorion (*m*/*z*)	Product Ion (*m*/*z*)	Declustering Potential (V)	Collision Energy (eV)
PFHpA	8.29	363	319, 169	−30, −30	14, 24
PFOA	9.25	413	369, 169	−40, −30	14, 24
PFNA	10.07	463	419, 219	−35, −35	16, 24
PFDA	10.75	513	469, 219	−40, −40	18, 26
PFHxS	8.38	399	79.9, 99	−90, −90	88, 72
PFOS	10.04	499	79.9, 99	−105, −105	106, 98
